# Exploratory study to examine the neuroendocrinological changes in typically developing adults during a music-related participatory practice using computer software

**DOI:** 10.3389/fpsyg.2024.1431952

**Published:** 2025-01-28

**Authors:** Yosuke Sugiyama, Sanae Tanaka, Aiko Komagome, Teruko Yuhi, Kazumi Furuhara, Haruhiro Higashida, Takahiro Tsuji, Mitsuru Kikuchi, Chiharu Tsuji

**Affiliations:** ^1^Public Collaboration Center, Tokyo University of the Arts, Tokyo, Japan; ^2^Research Center for Child Mental Development, Kanazawa University, Kanazawa, Japan; ^3^Division of Socio-Cognitive-Neuroscience, Department of Child Development, United Graduate School of Child Development, Osaka University, Kanazawa University, Hamamatsu University School of Medicine, Chiba University and University of Fukui, Kanazawa, Japan; ^4^Arts-Based Communication Platform for Co-Creation to Build a Convivial Society, Tokyo University of the Arts, Tokyo, Japan; ^5^Department of Ophthalmology, Faculty of Medical Sciences, University of Fukui, Fukui, Japan; ^6^Department of Psychiatry and Neurobiology, Graduate School of Medical Sciences, Kanazawa University, Kanazawa, Japan

**Keywords:** oxytocin, cortisol, group activity, workshop, stress, music, computer music, saliva

## Abstract

There has been a growing recognition of the benefits of participating in art practices for promoting well-being and social connection. Despite this, only a limited number of studies have assessed the neuroendocrinological changes that might contribute to these benefits. In this exploratory study, we focused on a creative activity related to music composition using digital tools. The emergence of computer software to create music (CSCM) has lowered the barriers to musical technical skills and theory, making music composition more accessible. We examined whether incorporating CSCM into a music-making workshop would affect the levels of two hormones, oxytocin and cortisol, among healthy adults. These two hormones were chosen, because oxytocin is involved in prosocial behavior and bonding, while cortisol plays a role in the stress response. Considering the time it takes to learn and adapt to a typical customized CSCM, we simplified its use to allow participants to experience music-making within a short timeframe and set up two distinct workshops. One was individual music creation with the support of a facilitator (Dyad) and the other was music creation in a group (Group). Participants in the Dyad workshops showed increased oxytocin levels, whereas those in the Group workshops did not. Cortisol levels remained unchanged during the Dyad workshops, but decreased in the Group ones. These results suggest that neuroendocrinological changes may occur during music-making activities using computer software. This work highlights the potential value of CSCM-incorporated music-making activities, although further controlled studies are required to confirm these findings.

## 1 Introduction

Over the past decade, there has been a growing recognition of the benefits of participating in music practices for promoting well-being and social connection (Williams et al., 2023; Estevao et al., 2021; Fancourt and Finn, 2019). Well-being refers to being physically healthy and emotionally fulfilled both within ourselves and in our relationships with others and our environment. Positive interactions with the people around us and satisfaction in our relationships promote a sense of well-being. These effects have been explored through qualitative methods, such as detailed process-focused analysis of interviews, focus groups, observational data, and questionnaires using non-standardized or standardized measures (Williams et al., 2023; Hacking et al., 2006; Hacking et al., 2008; O’Donnell et al., 2022). Positive impacts of participating in music activities on emotions, behaviors, and social connectedness have been documented in numerous studies (Williams et al., 2023; Calo et al., 2020; Perkins et al., 2020). For example, numerous reports indicate that group singing and drumming can increase self-esteem and self-confidence, reduce stress, improve mood, and decrease anxiety (Williams et al., 2023; Perkins et al., 2020). The DRUMBEAT program, which uses drumming as a way to engage at-risk youth, reported that school absenteeism and incidences of poor behavior decreased (Wood et al., 2013). Participants who engaged in music-based activities felt increased connectedness (Williams et al., 2023; Perkins et al., 2020; McNaughton et al., 2016; Southcott and Joseph, 2014). However, many reports that rely on qualitative analysis may be influenced by subjective judgments particularly when the analysis involves participant feedback and psychosocial questionnaires. There is thus a need for further interdisciplinary research incorporating both quantitative and qualitative assessments to strengthen our understanding of the outcomes of these practices.

Trust, social connectedness, the capacity to relate to others, and reduced stress are factors that promote well-being (Ishak et al., 2011). These factors may be linked to the neuropeptide oxytocin, which plays diverse roles in social recognition and behavior among mammals (Ishak et al., 2011). Numerous studies have reported that oxytocin promotes social affiliation and bond formation, reduces anxiety, and mitigates the stress response among mammals, including humans (Jurek and Neumann, 2018; Carter et al., 2020; Andari et al., 2018). Additionally, in humans, oxytocin plays an important role in attachment (Feldman et al., 2010; Feldman et al., 2010; Feldman et al., 2011), romantic relationships (Schneiderman et al., 2012), and attitudes or behaviors toward in-groups and out-groups (De Dreu, 2012; De Dreu et al., 2010). Oxytocin also fosters prosocial behaviors such as trust, empathy, and cooperation (Bartz et al., 2011).

In a previous study, we examined the neuroendocrinological response in children with autism spectrum disorder (ASD) and typically developing children attending a visual art-based participatory art workshop (Tanaka et al., 2020). We measured their salivary oxytocin and cortisol levels, with cortisol serving as a biomarker of the stress response. The hypothalamic–pituitary–adrenal (HPA) axis regulates the stress response, and dysfunction of this axis is associated with physiological stress (Ulrich-Lai and Herman, 2009). Elevated cortisol levels typically indicate HPA dysfunction and a stress response (Stoffel et al., 2021). Our findings revealed that the increase in salivary oxytocin levels following art activities performed by children with ASD was significantly greater than that observed in typically developing children. However, neither group exhibited any change in the salivary cortisol levels. These results suggest that the beneficial effects of participatory art activities may be partially mediated by oxytocin release and could hold therapeutic potential for disorders characterized by social dysfunction. Therefore, salivary oxytocin and cortisol levels may be valuable as markers for assessing the beneficial effects of arts-based practices.

The integration of digital technology into music-related practices has been gaining popularity for decades (Emmerson, 2018). Here we focus on creative activity involving music composition using computer software. Employing digital tools often facilitates the achievement of the main goals of the activities. For instance, in the “Turn your iPhone into a Sensor Instrument!” workshop, participants learned to perform music using “instrumentalized’ iPhones (Parkinson and Tanaka, 2018). Real-time digital synthesis is achieved through RjDj, an app launched on the iPhone that allows “patches” to run which is authorized with Pure Data, the visual programming environment (Parkinson and Tanaka, 2018). Participants quickly grasped the basics, demonstrating that digital tools were effective for introducing music performance to people without prior musical experience (Parkinson and Tanaka, 2018). Although music composition was once considered a highly creative process that benefits many, it has historically been challenging due to the need for a deep understanding of music theory and prior musical experience (Craw, 2006; Kennedy, 2002). However, the introduction of computers, particularly sophisticated software like the Digital Audio Workstation (DAW), has revolutionized the music landscape by removing barriers to music technical skills and theory (Bennett, 2018; Bell and Ratcliffe, 2015). Notably, since the late 1990s, DAWs have been incorporated into educational settings to enhance music education (Black and Wilson, 2024; Reynolds, 2005; Dobson and Littleton, 2016; Brown and Brown, 2012). The development of user-friendly software, for instance, GarageBand, Ableton Live, and/or Soundtrap, along with the increasing availability of computers and the internet in schools, has made it possible for educators to introduce DAW into music education (Knapp et al., 2023; Uludag and Satir, 2023; Sabet, 2020; Burnard, 2012; Özer and Demirbatir, 2023). DAW facilitates students’ understanding of music composition, production, and theory by providing hands-on experience with recording and editing software (Brown and Brown, 2012). These tools enable students to create, arrange, and manipulate music digitally, fostering creativity and technical skills (Brown and Brown, 2012). The development of creative thinking through music production using DAW has also been reported (Yanan, 2024; Cheng, 2023).

In this study, we examined whether neuroendocrinological changes occur during music-making workshops using computer software in typically developing adults. To address the time it takes to learn and adapt to a typical customized DAW, we simplified the use of music-making software during the workshop and set up two distinct workshops. The first involved individual participants creating music with the assistance of a facilitator (Dyad workshop). The second involved groups of subjects collaboratively creating music (Group workshop). Saliva samples were collected from participants during the workshop to examine changes in oxytocin and cortisol levels. To the best of our knowledge, this is the first exploratory study to investigate oxytocin and cortisol changes in adults participating in music creation workshops utilizing computer software.

## 2 Materials and methods

### 2.1 Participants

Overall, 12 (2 men, 10 women) and 17 (3 men, 14 women) typically developed adults participated in the Dyad and Group CSCM workshops, respectively. Of these participants, 11 adults (2 men, 9 women) aged between 19 and 50 years old (mean age: 39) and 13 adults (3 men, 10 women) aged between 19 and 54 years old (mean age: 35) were assessed for salivary oxytocin and cortisol levels for the Dyad and Group workshops, respectively (Table 1). Of these subjects who underwent saliva measurements, 8 participated in both Dyad and Group workshops. Additionally, 1 participated first in Dyad workshops and then in Group ones, while in 7 the opposite order was followed. Each participant experienced a Dyad and/or Group workshop only once.

**Table 1:** Demographic features of participants who underwent salivary measurements.

	Number of Participants	Age	Anxiety traits	State anxiety	SDS row score	Average of QOL
All	24	37.1 ± 2.62	43.83 ± 2.084	42.00 ± 2.522	39.25 ± 1.142	3.56 ± 0.090*
Dyad	11	39.4 ± 3.50	43.73 ± 2.727	44.18 ± 3.252	40.09 ± 1.856	3.55 ± 0.144
Group	13	35.1 ± 3.86	43.92 ± 3.181	40.15 ± 3.799	38.54 ± 2.147	3.57 ± 0.123*

*Average of QOL of All, *N* = 23. Average of QOL of Group, *N* = 12.

### 2.2 Questionnaires

Participants completed the State–Trait Anxiety Inventory (STAI) for state anxiety and trait anxiety, the Self-rating Depression Scale (SDS), and personal questionnaires. The STAI and SDS questionnaires were posted to participants to complete at home and they brought them back with them on the day of the workshop. None of the participants scored above the threshold for anxiety or depression on the traits measure and SDS scores (Table 1). Personal questionnaires including questions on basic demographics (sex, date of birth), physical and mental health status, smoking habits, medication, use of hormonal contraceptives, stress, preceding physical activity, and the last time they consumed food were administered to assess the factors influencing the hormonal measurements. Participants completed the personal questionnaires when they arrived. The exclusion criteria for undergoing analysis of saliva samples included self-reported chronic medication usage, psychiatric conditions, and health issues (e.g., Lifestyle related diseases). Before and after the workshops, the participants completed a visual analog scale (VAS) to describe their emotions. The VAS consisted of vertical lines, each 100 mm in length, with anchor statements at the top and bottom. The questions assessed happiness, vitality, liking or disliking the art activity in general, relaxation, and tension. Additionally, after the workshops, the participants were asked whether felt satisfied, had had fun, or considered the activity to be a favorite of theirs. Higher scores indicate more positive responses.

### 2.3 Ethics statement

This study was approved as a noninvasive medical study by the ethics committee of Kanazawa University Graduate School of Medicine in 2018 (approval number #2018–025). The study was conducted in accordance with the tenets of the Declaration of Helsinki and the Ethical Guidelines for Clinical Studies of the Ministry of Health, Labour, and Welfare of Japan. After the participants had been given a complete explanation and written information about the study, they voluntarily provided written informed consent. The participants were informed that they could choose not to supply their saliva on each occasion, even after agreeing to participate in the study.

### 2.4 Music creation workshop utilizing computer software

Two kinds of workshops that ustilize computer software to create music (CSCM-workshop) were conducted in this study. One was a workshop with individuals, each of whom had a facilitator support (Dyad). Participants made music throughout the activity. The other involved groups of 4 people or more making music together (Group). Each workshop was conducted by the same facilitator. The workshops were conducted either at Kanazawa University or at Tokyo University of the Arts. To avoid differences caused by variations in the environment, the workshops took place in rooms of similar sizes with dark-colored carpeting and white walls. The participants were instructed to avoid eating, drinking other than water, brushing their teeth, or engaging in any physical activities for at least 1 h before attending the workshop. The participants arrived 20 min before the workshop started and were seated in the workshop room. In both cases, participants were seated in the workshop room, with all Group participants waiting together in the same room. When they arrived, we first asked them to drink some water if needed and then if possible, to avoid drinking water during the workshop. However, if they needed to, we asked them to drink just after sampling. The participants received an explanation of the workshop and provided written informed consent. Then participants fill out the VAS and personal questionnaire. The start time of the workshop and the number of participants who participated in each time window are shown in Supplementary Table 1. In brief, the Dyad workshops started at 10:30, 11:00, 13:00, 14:00, 14:45, and 16:45, while the Group workshops started at 11:00, 13:10, and 13:30. The Group workshops were held 4 times.

#### 2.4.1 Flow of the workshops

**Figure 1:**
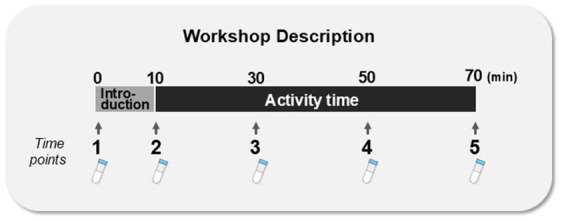
Saliva sampling points during workshops. Both Dyad and Group workshops consisted of 10 min of introduction and 60 min of activities.

Both the Dyad and Group workshops lasted 70 min and consisted of two parts: a 10-min introduction and 60-min music-making activities (Figure 1).

##### 2.4.1.1 Dyad workshop

**Figure 2:**
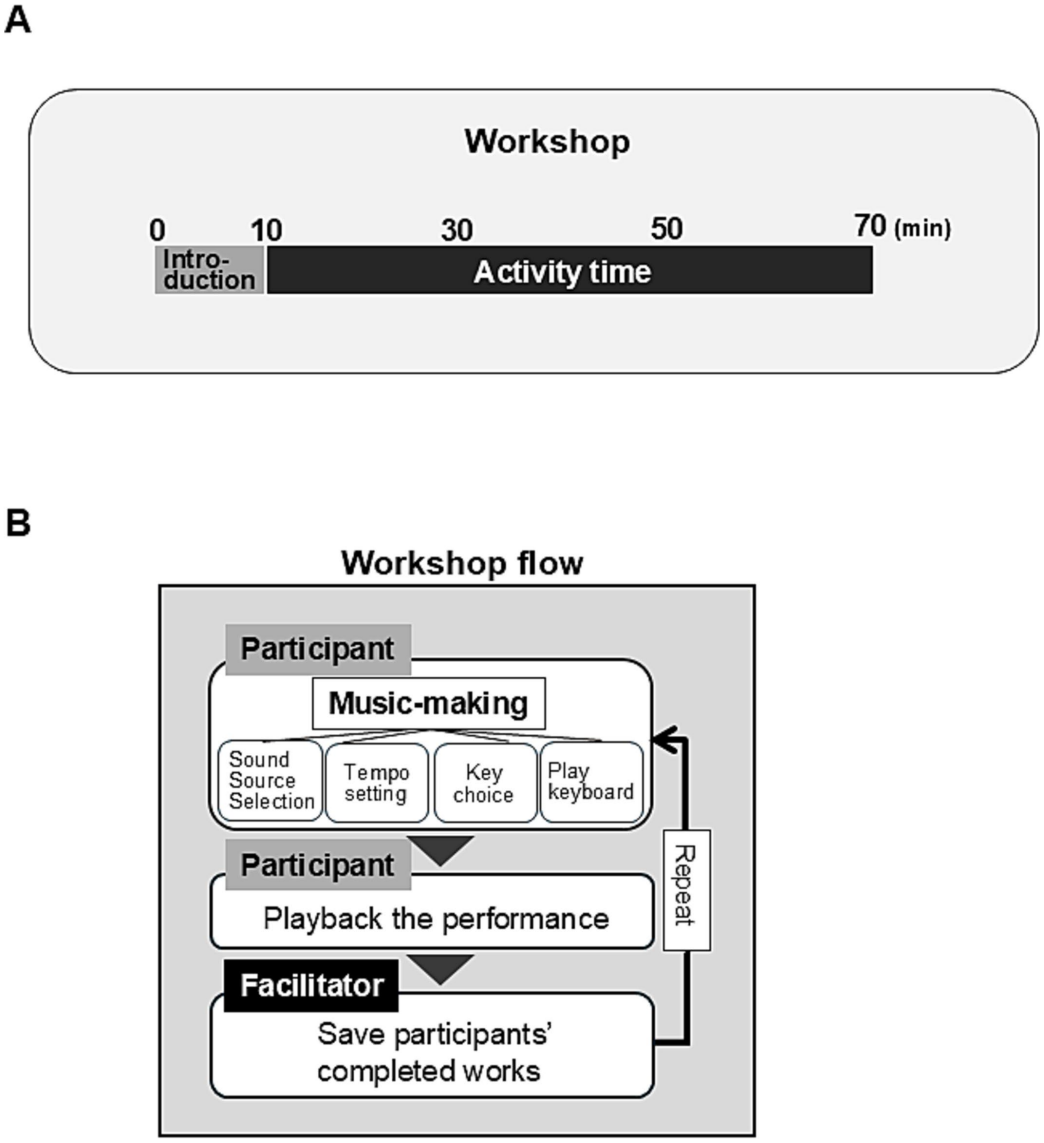
The workshop flow. **(A)** The time frame of the Dyad workshop. **(B)** The flow of the Dyad workshop. The computer software to create music (CSCM) in the facilitator’s personal computer was mirrored on an Apple Inc.’s iPad. However, the functions on the iPad could be restricted by the facilitator using the CSCM on the computer. For participants to concentrate on music-making on the iPad, the iPad’s functions were limited to sound selection, tempo setting, key choice, and playing notes. During the activity, participants repeatedly created 4-bar music pieces with the facilitator providing support as needed. The facilitator used the CSCM on the computer to record and play back the music that the participants had made on the iPad.

During the activity part of the Dyad workshop, the participants repeatedly created 4-bar music pieces with support from the facilitator provided as needed (Figure 2).

##### 2.4.1.2 Group workshop

**Figure 3:**
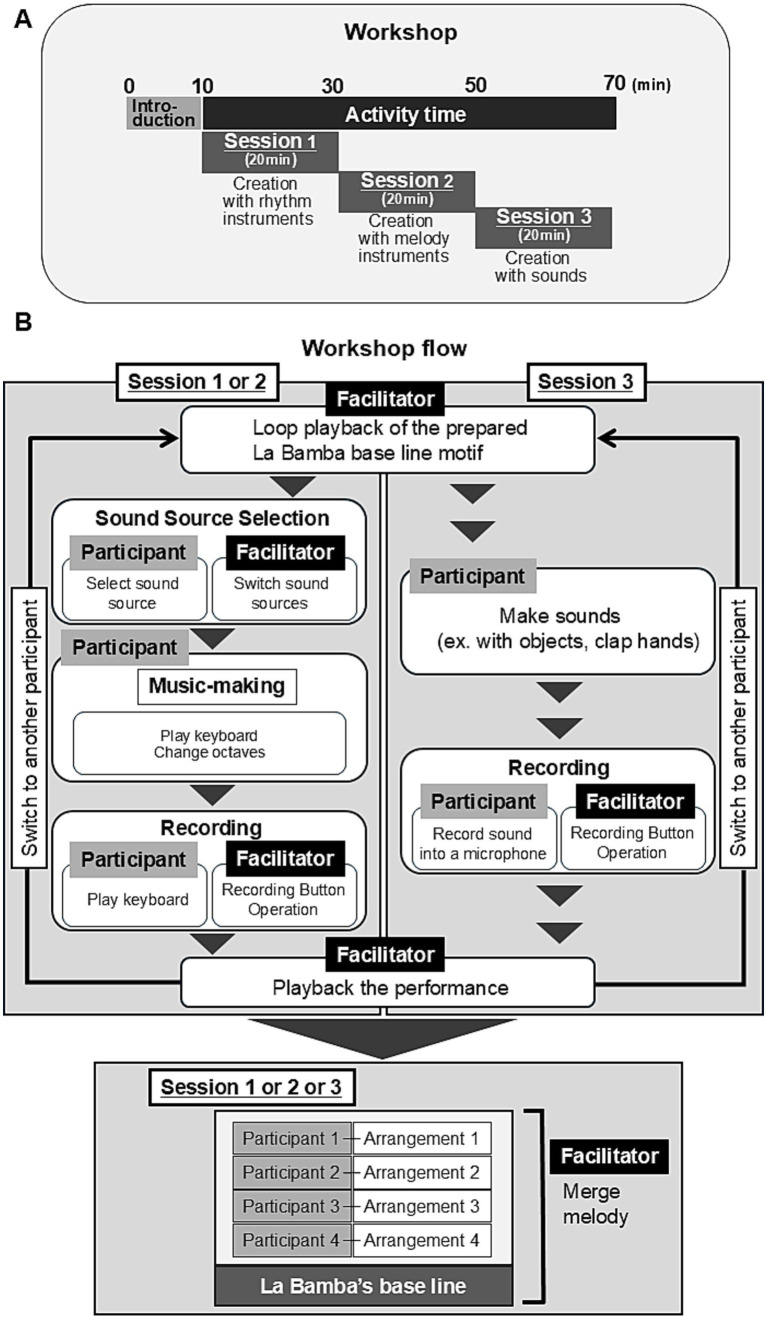
Flow and time frame of the Group workshop. **(A)** The activity part was divided into three connected sessions without any breaks between them. **(B)** The software, Logic Pro, was installed on the facilitator’s computer. Participants made a melody using 18 notes with a keyboard connected to the computer. The pitch could be changed on the keyboard. There are approximately 100 sound options (mirroring various instruments) in Logic Pro, so the participants could choose the sound they wanted to make music with. Participants told the facilitator the choice that they made and the facilitator switched the sound source in Logic Pro. Once the participants had created a sequence of notes or sounds to accompany the “La Bamba” tune, the facilitator recorded the music. The facilitator then merged and played back all of the music created by the participants along with the “La Bamba” tune.

The activity in the Group workshop was divided into three connected sessions without any breaks between them (Figure 3).

Session 1: each participant created a sequence of notes using rhythmic instruments that aligned with the baseline of “La Bamba.” These rhythms were recorded by the facilitator.

Session 2: the participants used melody instruments to create sequences of notes that aligned with the baseline, which were also recorded.

Session 3: the participants created sounds using their voices or objects that aligned with the baseline. These were also recorded.

In each session, all recorded sequences of notes or sounds were merged and played together to the tune of “La Bamba” by the facilitator.

#### 2.4.2 Criteria for choosing a baseline tune for the group workshop

The participants were required to create original melodies and rhythm patterns within a short timeframe. To facilitate their creative activities and ensure they could quickly grasp the tasks involved in the activity, it was necessary to select a musical piece for the baseline that met the following conditions.

Presenting motifs with minimal measures: the basic motif should be presented in a minimal number of measures.

Presenting low-range motifs: the motif should be in a lower range to serve as an accompaniment, making it easier to layer melodies and rhythm patterns on top of it.

Simple key: the piece should be in a simple key to reduce complexity during creation.

Loop playback of motifs: it should be possible to loop the motif continuously without disrupting participants’ creative thoughts, ensuring that it benefits all participants, even those in the middle of the creative process. This continuous loop would simultaneously help to stimulate creative ideas.

High versatility in arrangement: the piece should have a proven record of being adapted into various musical forms, like popular songs or wind ensembles. This would suggest that the participants could easily create unique arrangements using it.

Based on these criteria, we chose the “La Bamba” baseline. This further meets the applied requirements by using a 2-bar pattern repeated 4 times, making a total of 8 bars, and being in the C major scale.

#### 2.4.3 Devices used in the workshop and the roles of the facilitator and participants

##### 2.4.3.1 Dyad workshop

**Figure 4:**
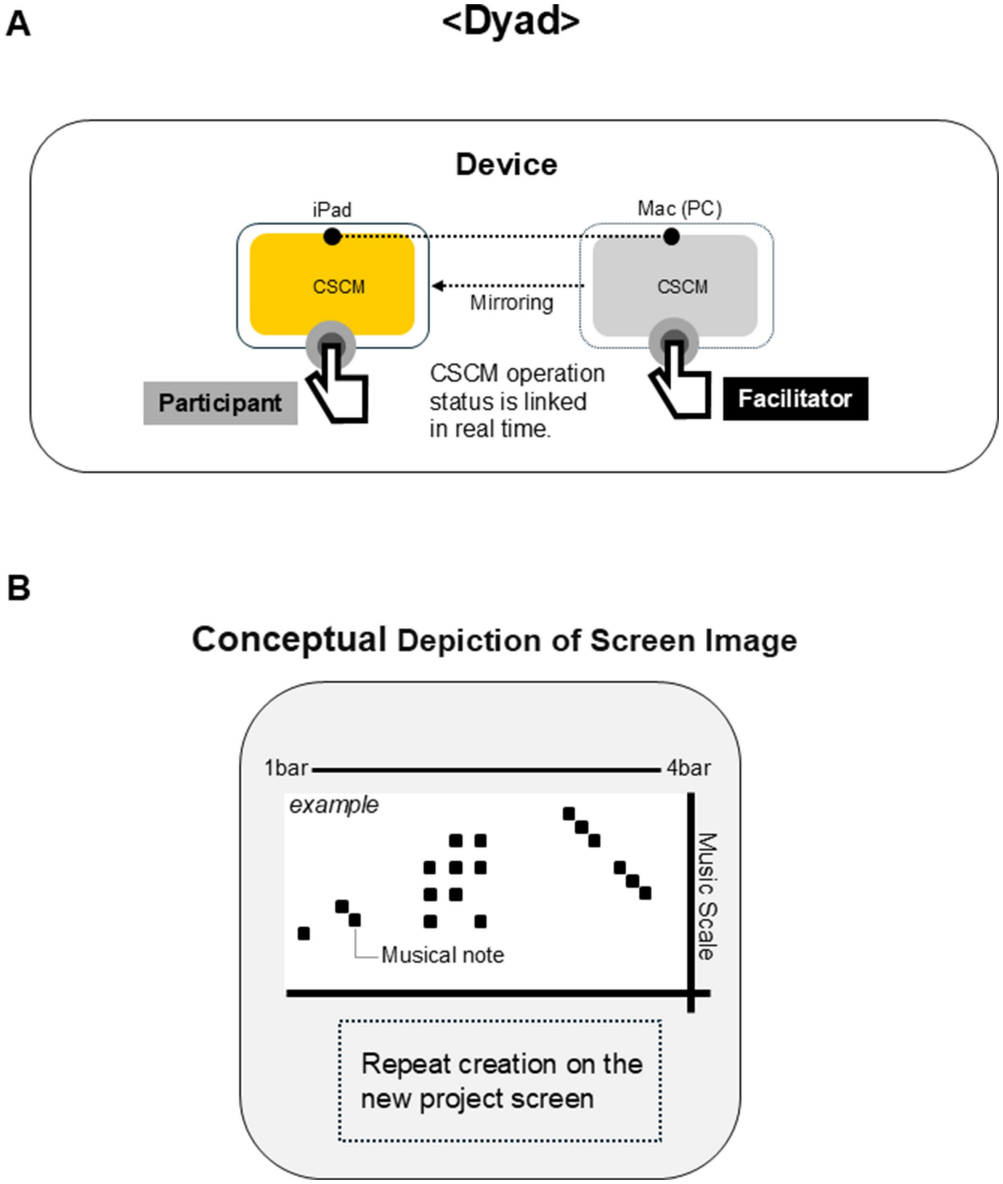
Conceptual depiction of the device used in the Dyad workshops. **(A)** Participants used an Apple Inc.’s iPad to make music, which was linked to the software to create music (CSCM) in the computer controlled by the facilitator. **(B)** Conceptual depiction of the screen image of iPad.

A computer software to create music (CSCM) was developed using Max (Cycling ‘74, Covina, CA, USA) to allow the participants to experience music composition in a short timeframe. The CSCM was launched by the facilitator’s computer on which Max was installed. CSCM was mirrored to an iPad (Apple, Inc., Cupertino, CA, USA) for use by the participant, but the functions in the iPad could be restricted by the CSCM on the computer. The functions on the iPad were limited to sound selection, tempo setting, key choice, and playing notes during the workshop. Participants engaged in trials to create music by typing notes using an iPad. When they felt satisfied, they proceeded with the final playing. The facilitator used the CSCM on the computer to record and play back the music that the participant had created. The conceptual depiction of the device is shown in Figure 4.

##### 2.4.3.2 Group workshop

**Figure 5:**
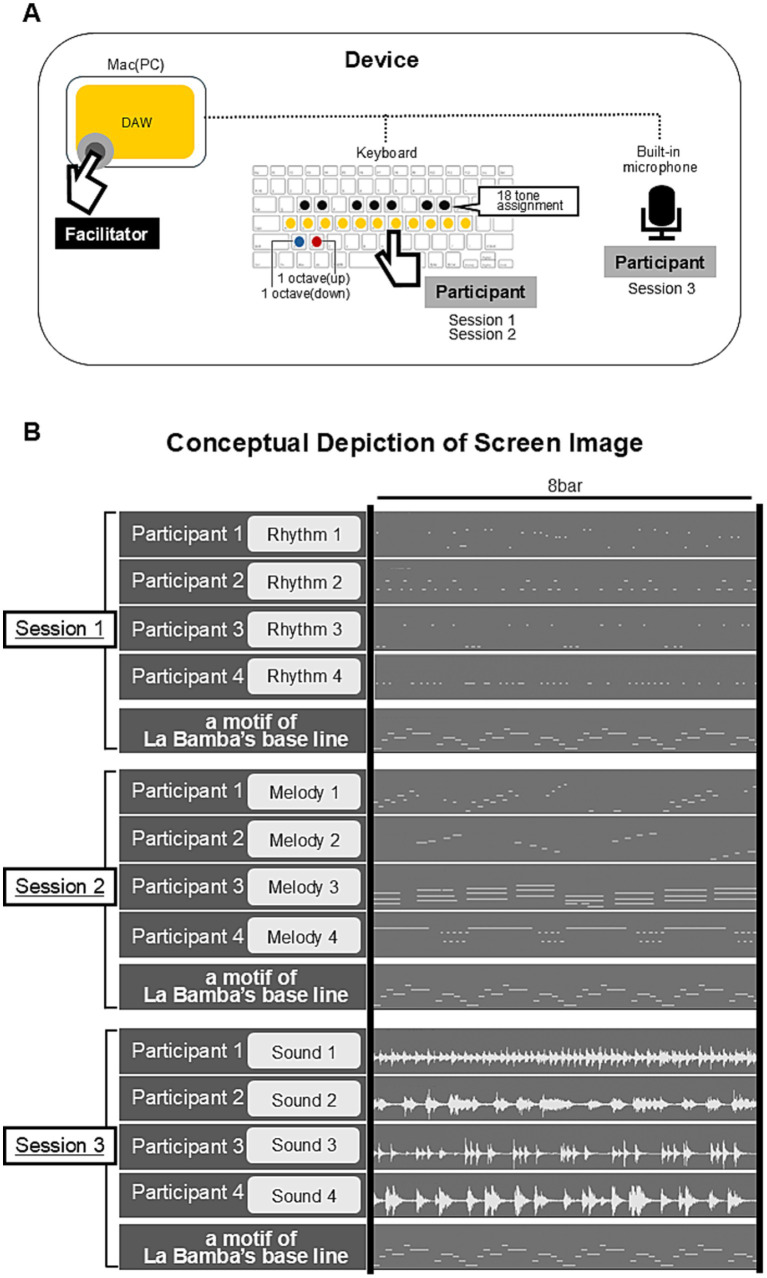
Conceptual depiction of the device used in the Group workshops. **(A)** Participants used a keyboard connected to Logic Pro in the computer controlled by the facilitator. In sessions 1 and 2, the participants made music with the keyboard. In session 3, the participants made sounds and were recorded through a built-in microphone on the computer. **(B)** Conceptual depiction of the screen image on the computer. The screen image was shared with participants via a projector, visualized on a large screen. An example of the three sessions during the activity. In the first session, participants selected different rhythmic instruments, in the second session they selected melodic instruments, and in the third session, they made sounds, for example, clapping their hands or making sounds using objects. Participants engaged in trials to create music by typing notes on the keyboard or producing sounds. When they felt satisfied, they proceeded with the final playing, and the facilitator recorded their music.

The computer software used in the Group workshop was Logic Pro (Apple, Inc.) and was installed on the facilitator’s computer. Logic Pro was selected for its extensive selection of sound options and sound quality. A keyboard was connected to the computer and the participants used it to make a melody using 18 notes. The pitch of the keyboard could also be changed. There are approximately 100 options for sounds (mirroring various instruments) in Logic Pro and the participants told the facilitator which sound they wanted to make music with. Then, the facilitator switched the sound source in Logic Pro. Participants engaged in trials to create music by typing notes on the keyboard or producing sounds. When they felt satisfied, they proceeded with the final playing, and the facilitator recorded their music. The facilitator then merged and played together to the tune of “La Bamba.” The conceptual depiction of the device is shown in Figure 5.

### 2.5 Saliva collection and analysis

Saliva samples were collected five times: just before the start of the workshop, after the introduction, every 20 min during the activities, and finally at the workshop’s end (Figure 1). The saliva samples (1.0–1.5 mL) were collected in a sterile 15 mL polypropylene tube (Greiner Bio-one Co. Ltd., Tokyo, Japan) and immediately placed on ice. After the workshop was over, the samples were stored at −20°C. The participants were asked to contact us if they developed any contagious disease within 2 weeks after the workshop. Those saliva samples from the participants who did not report an infection within the 2-week period were thawed in a draft chamber and centrifuged at 4°C and 1,500 × g for 10 min. The samples were then divided into 1.5-mL microtubes, each containing 100 μL, and frozen again at −80°C until being used for assaying. The salivary oxytocin level was measured using a 96-well plate commercial oxytocin-ELISA kit (Enzo Life Sciences, Farmingdale, NY, USA), as described previously (Tanaka et al., 2020; Minami et al., 2022; Tsuji et al., 2015). The measurements were performed in duplicate. Samples (100 μL) not subjected to fractionation were treated in accordance with the manufacturer’s instructions. The optical density of the samples and standards was measured at a wavelength of 405 nm by a microplate reader (Bio-Rad, Richmond, CA, USA). The salivary cortisol level was measured using a cortisol enzyme immunoassay kit (Salimetrics, State College, PA, USA), as previously described (Tanaka et al., 2020). Samples (25 μL) were treated in accordance with the manufacturer’s instructions. The optical density of the samples and standards was measured at a wavelength of 450 nm by a microplate reader (Bio-Rad, Richmond, CA, USA). Measurements were performed in duplicate. Sample concentrations were calculated according to the relevant standard curve. The intraassay and interassay coefficients were < 7.5% and < 8.1%, respectively.

### 2.6 Statistical analysis

Statistical analysis was performed by one-way repeated measures ANOVA and Student’s t-test using Prism 8 software (GraphPad Software Inc., San Diego, CA, USA). The relative values of salivary oxytocin and cortisol at each time point were determined by dividing the value of that time point by the level at just before the start of the workshop. All data were calculated as mean ± standard error of the mean. In all analyses, *p* < 0.05 was considered statistically significant.

## 3 Results

**Figure 6:**
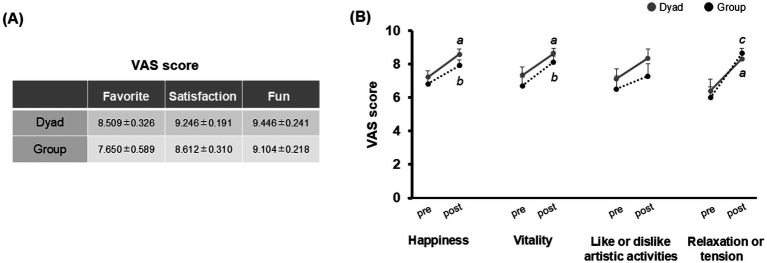
Emotional state is measured with the visual analog scale (VAS). **(A)** Participants’ responses after the CSCM workshop. **(B)** VAS scores before (pre) and after (post) the Dyad and Group CSCM workshops. Significant differences between pre and post at: Dyad, ^a^*p* < 0.05; Group, ^b^*p* < 0.05, ^c^*p* = 0.001.

We assessed the emotional state of the participants using the VAS (Figure 6). The scores obtained upon asking how the participants felt after experiencing the workshop were relatively high for both workshops. Higher scores corresponded to more positive feelings, indicating that the participants had a positive impression of the workshop (Figure 6A). Additionally, post emotional scores on happiness, vitality, and relaxation or tension, were significantly higher than pre scores in both Dyad and Group participants (Dyad and Group, happiness and vitality, *p* < 0.05, Dyad, relaxation or tension, *p* < 0.05, Group, relaxation or tension, *p* = 0.001, Figure 6B).

**Figure 7:**
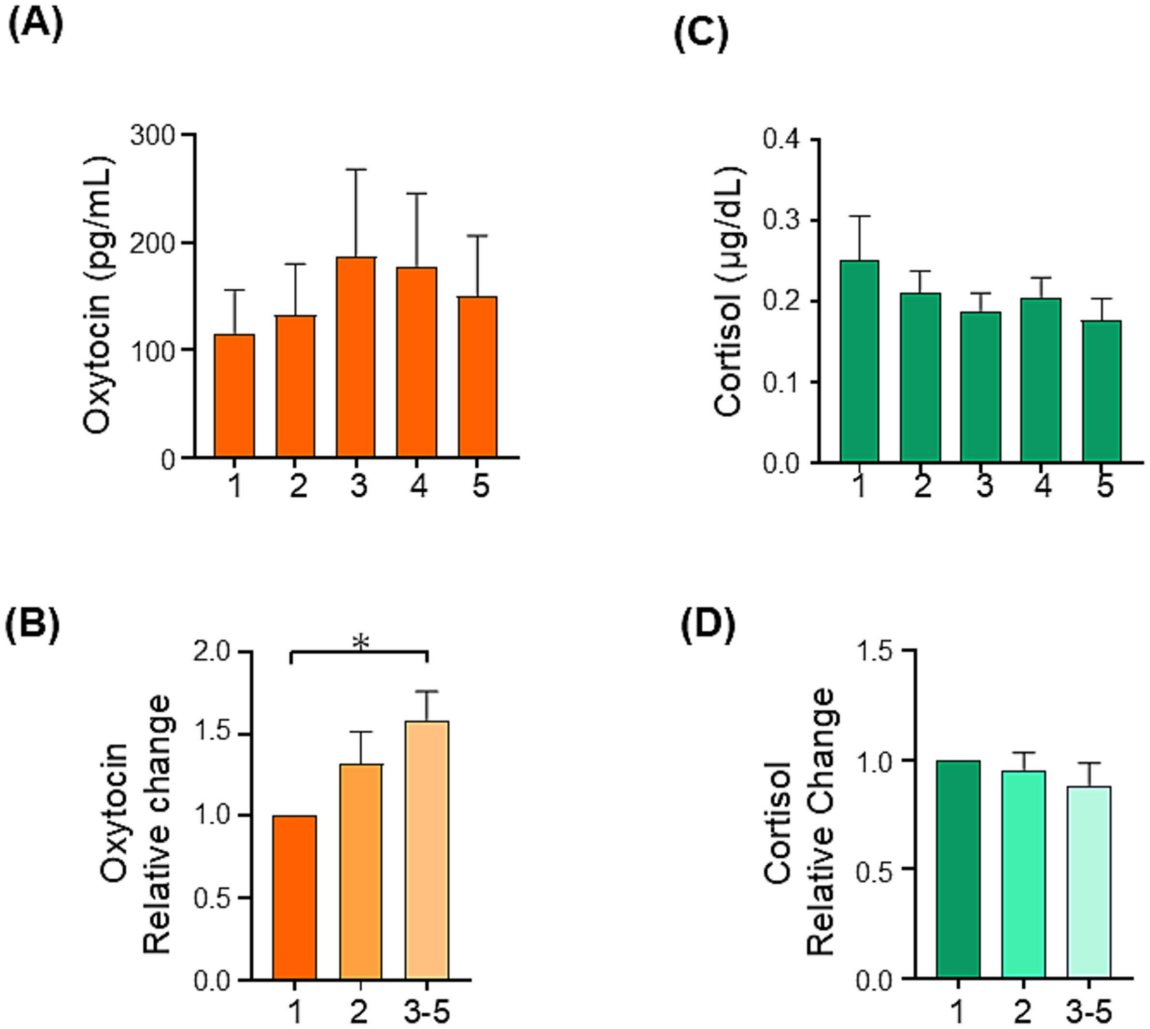
Changes in salivary oxytocin and cortisol levels during the Dyad CSCM workshop. **(A)** Salivary oxytocin level during the CSCM workshop. **(B)** Relative changes in salivary oxytocin. **(C)** Salivary cortisol level during the CSCM workshop. **(D)** Relative changes in salivary cortisol. Eleven adults were analyzed. Data are mean ± sem. *p < 0.05.

In the Dyad workshop, the oxytocin concentration tended to increase during the activity part, but this did not reach significance (Figure 7A). Next, we analyzed the relative change in the oxytocin level during the workshop. Owing to the variability in participants’ peak oxytocin levels during the activity itself, we averaged the relative values from the third to fifth time points. RM-ANOVA revealed significant differences [*F* (1.930, 19.30) = 4.609, *p* = 0.024; Figure 7B], and the *post hoc* analysis using Tukey’s multiple comparison test showed significant differences in this variable between the first and the average of the third to fifth timepoints (*p* = 0.019). Given the higher number of female participants, we further analyzed the data from female participants only. The relative change in oxytocin level in only females from the first time point to the average of the third to fifth time points was found to be significant using the Student’s *t*-test (*p* = 0.037, Table 2). Meanwhile, no significant differences in the absolute and relative value of cortisol were identified (*p* = 0.131, Figures 7C,D). In summary, the levels of oxytocin were increased during Dyad workshop, but no such significant change in the cortisol level was observed.

**Table 2:** The absolute values of oxytocin (OT) and cortisol (CORT), along with their relative rates from the first time point to the average of the third to fifth time points, are presented.

Dyad	Number of participants	Absolute value (time point 1)	Relative change ratio (1 vs 3–5 average)	*p* value (1 vs 3–5)
OT	Male/Female (2/9)	114.4 ± 41.13 pg/mL	1.585 ± 0.175	0.007**
	Female (9)	96.2 ± 40.85	1.514 ± 0.206	0.037*
	Female (5)-AM-	107.8 ± 67.08	1.353 ± 0.173	0.111
	Female (4)-PM-	81.7 ± 48.86	1.715 ± 0.425	0.191
CORT	Male/Female (2/9)	0.250 ± 0.055 μg/dL	0.879 ± 0.106	0.280
	Female (9)	0.263 ± 0.067	0.815 ± 0.110	0.131
	Female (5)-AM-	0.193 ± 0.052	0.816 ± 0.131	0.232
	Female (4)-PM-	0.350 ± 0.135	0.814 ± 0.210	0.442

Group	Number of participants	Absolute value (time point 1)	Relative change ratio (1 vs 3–5 average)	*p* value (1 vs 3–5)
OT	Male/Female (3/10)	131.0 ± 37.67 pg/mL	1.427 ± 0.232	0.091
	Female (10)	126.3 ± 47.51	1.539 ± 0.280	0.086
	Female (2)-AM-	74.4 ± 47.95	0.845 ± 0.163	―
	Female (8)-PM-	139.2 ± 58.53	1.713 ± 0.322	0.062
CORT	Male/Female (3/10)	0.235 ± 0.043 μg/dL	0.715 ± 0.096	0.012*
	Female (10)	0.236 ± 0.055	0.696 ± 0.119	0.030*
	Female (2)-AM-	0.190 ± 0.042	0.704 ± 0.151	―
	Female (8)-PM-	0.248 ± 0.068	0.693 ± 0.148	0.077

The number of participants who underwent salivary measurements, along with their sex, is noted. Due to the higher number of female participants, statistical analysis was conducted. Additionally, the group of female group was further analyzed based on the start time of the workshop, either AM or PM. Values are presented as mean ± SEM. Student’s *t*-test, *p < 0.05, **p < 0.01.

**Figure 8:**
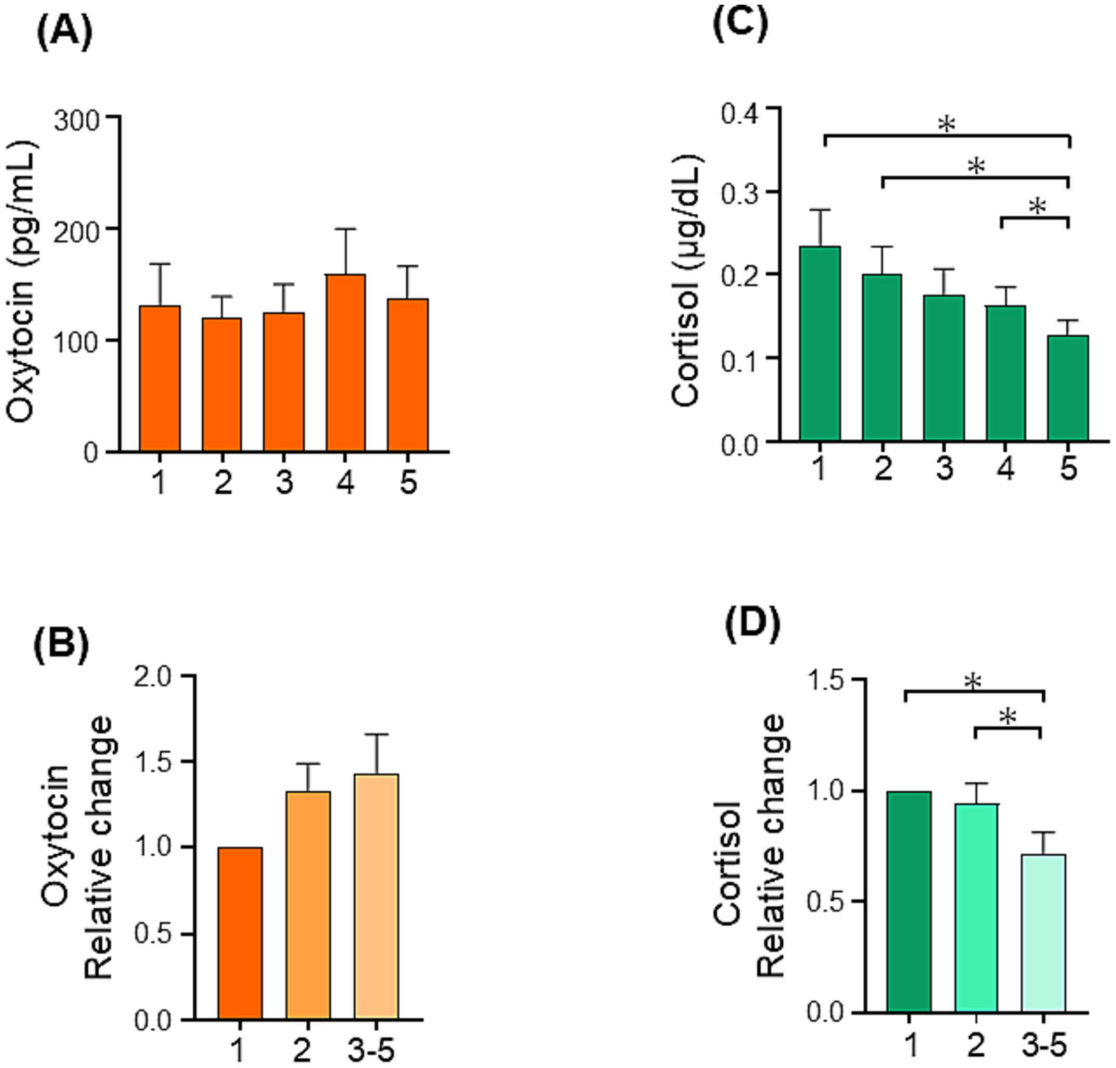
Changes in salivary oxytocin and cortisol levels during the Group CSCM workshop. **(A)** Salivary oxytocin level during the CSCM workshop. **(B)** Relative changes in salivary oxytocin. **(C)** Salivary cortisol level during the CSCM workshop. **(D)** Relative changes in salivary cortisol. Thirteen adults from four workshops were analyzed. Data are mean ± sem. *p < 0.05.

In the Group workshop, there was a tendency for oxytocin levels to increase, but no significant differences in both absolute and relative values were observed among the time points (Figures 8A,B). However, cortisol levels showed a significant difference among the timepoints [RM-ANOVA, *F* (1.592, 19.11) = 8.084, *p* = 0.005; Figure 8C]. *Post hoc* comparisons revealed significant differences in this variable between the first and fifth timepoints (*p* = 0.037), second and fifth timepoints (*p* = 0.023), and fourth and fifth timepoints (*p* = 0.015). The relative value of cortisol demonstrated a significant decrease as well [RM-ANOVA, *F* (1.849, 22.19) = 5.844, *p* = 0.01; Figure 8D]. Post hoc comparisons revealed significant differences between the average of the third to fifth timepoints and the average of the first (*p* = 0.03) and second timepoints (*p* = 0.027). Additionally, considering the predominance of female participants, we further analyzed the data for female participants only. Upon comparing the relative change in oxytocin level from the first time point to the average of the third to fifth time points using Student’s *t*-test, a trend for an increase was identified (*p* = 0.086, Table 2). Meanwhile, a significant decrease in the cortisol level was observed (*p* = 0.03). In summary, our results for the Group workshop showed that oxytocin levels had a tendency to increase while cortisol levels exhibited a significant decrease.

## 4 Discussion

The objective of the current trial was to exploratively examine the neuroendocrinological changes in healthy adults participating in workshops that ustilize computer software to create music (CSCM, CSCM workshop). During the Dyad workshops, oxytocin levels significantly increased, while only a tendency for such an increase was shown in the Group workshops. Meanwhile, cortisol levels remained unchanged during the Dyad workshops, but decreased in the Group workshops. These findings suggest that neuroendocrinological changes may occur during the CSCM workshops.

It is important to emphasize the distinctions between the two types of workshops explored here. Considering the time it takes to learn and adapt to a typical customized DAW, we simplified its use to allow participants to experience music-making within a short timeframe and set up two distinct workshops. The purpose of this study was to exploratively examine whether subjects participating in CSCM workshops show neuroendocrinological changes, rather than to directly compare the participation types, namely, groups or dyads. Further studies with comparable experimental conditions, such as using the same kind of music-creation software for both Group and Dyad activities, are necessary to examine the correlation between neuroendocrinological changes and the contents of such workshops.

Considering the differences in the Dyad and Group workshops, one possible factor influencing the discrepant findings regarding oxytocin is the differences in the level of communication during them. Compared with the Dyad activities, there was less communication and conversation among participants or between participants and facilitators during the group activities. The duration during which each participant spoke accounted for approximately 10 and 25% of the total conversation during the Group and Dyad workshop activity sessions, respectively. Another possible factor affecting the oxytocin response is the time spent making music during the activity sessions. In the Dyad workshops, the participants were engaged in composing music throughout the session, while in the Group workshops, they took turns creating music, for approximately 10 min per participant. Consequently, significantly less time was dedicated to music creation in the Group workshops than in the Dyad ones. Nonetheless, there is a need for further examination of the effects of workshop content and environmental or situational factors on oxytocin levels.

Salivary oxytocin did not increase during the Group CSCM workshops. Previous studies examining various positive social experiences such as parent–child interactions (Feldman et al., 2010; Feldman et al., 2011; Minami et al., 2022), massages (Tsuji et al., 2015), gossiping with friends (Brondino et al., 2017), and affectionate interactions between owners and their dogs (Nagasawa et al., 2015) have suggested that salivary oxytocin levels typically rise during such interactions. However, it appears that merely gathering together with unrelated individuals and engaging in a positive activity does not necessarily stimulate oxytocin release. Schladt et al. (2017) also investigated salivary oxytocin and cortisol levels during a positive social activity, namely, singing in a choir, and found that both oxytocin and cortisol levels decreased. One possible explanation for the lack of an increase in oxytocin levels in our study is the level of participant connectivity. As discussed by Schladt et al. (2017), bilateral bonding between participants may be necessary for a noticeable increase in oxytocin during positive social activities. Similarly, other reports have indicated that oxytocin tends to increase in situations involving close bonding, such as between parents and children, romantic partners, and dogs and their owners. This is consistent with our findings given that participants in our Group workshop either met for the first time or were not particularly close to each other. Furthermore, Schladt et al. (2017) reported that stress arousal, rather than social experiences, can increase oxytocin levels in saliva. While both cortisol and oxytocin levels decreased in their study, we observed a decrease only in cortisol levels and no significant change in oxytocin during the Group workshop. This discrepancy may stem from differences in the stress-inducing nature of the situations. While singing in a choir in the previous study occurred in public, our workshop setting was relaxed throughout. The significant decrease in cortisol levels observed in the choir study in contrast to the findings in our study suggests that fluctuations in oxytocin levels may be influenced by fluctuations in cortisol levels.

No increase in oxytocin was observed during the group activity in this study. Participants in this experiment were either strangers or acquaintances but not close to each other, making them out-group members. Oxytocin plays a vital role in social cognition and affects prosocial behavior, empathy, and the perception of facial expressions (Bartz et al., 2011). However, the impact of oxytocin on social cognitive processes depends on the context, such as the presence or absence of in-group or out-group members (De Dreu, 2012; De Dreu et al., 2010; De Dreu and Kret, 2016). Therefore, it is possible that the oxytocin response to group activities varies depending on the relationship among the participants, such as whether or not they are friends. Further experiments are needed to confirm the effects of in-and out-group dynamics in this context.

We observed a decrease in cortisol levels during the Group workshop. The cortisol level follows a circadian rhythm, peaking approximately 30 min after waking and thereafter declining during the day until it reaches its nadir during sleep (Weitzman et al., 1971). Aware that the cortisol level varies by sex and age, we took into account the data reported by Stoffel et al. (2021) between 5 and 8 h after waking up, salivary cortisol levels decreased by approximately 1 nmol/L per hour (i.e., 0.036 μg/dL per hour). During the 70-min Group workshop, the concentration of cortisol dropped from 0.235 to 0.129 μg/dL (Figure 8C). This decline appears to be greater than the expected decrease under the circadian rhythm. However, it is important to consider that the time window of our workshops was not consistent. The start time of the Group workshop varied within a 2-h window, starting between 11:00 to 13:30, and the sex and age of the participants were not matched. To validate the obtained findings, there is thus a need for further studies with participants of the same sex and similar age, as well as consistent workshop timing.

In this study, saliva samples were collected three times during the activity part to examine how oxytocin concentration changes over time. The results showed individual variations in the pattern of oxytocin levels during activity time. With this variable, no statistically significant difference was detected between the first time points and at any specific time point during the activity session. However, there was a tendency for oxytocin levels to increase during the activity, particularly in the Dyad workshop. Therefore, we calculated the average rate of increase across the three sampling timepoints and compared it to the first time points. In experiments investigating changes in oxytocin concentrations in response to “real-life” situations rather than fixed experimental stimuli, increasing the number of sampling points may be crucial to account for individual differences.

Our study had several limitations. First, the sample size was small, and the participants’ sex and age were not matched. Further studies with larger sample sizes and participants of the same sex and similar age groups are necessary to examine the correlation between neuroendocrinological changes and CSCM workshops. Second, we were unable to conduct the workshops within a consistent timeframe that takes into account the circadian rhythm. We should address the changes in cortisol levels during the CSCM workshops within a consistent time window. Third, we did not have comparable conditions to fully assess how social interaction in the Dyad condition and closeness in the Group condition affected the oxytocin response. Further studies are needed to understand the correlation between the oxytocin response and the contents of CSCM workshops with more specific comparable conditions. Finally, we used ELISA to measure saliva oxytocin concentrations, and our detected oxytocin concentration range was higher than that reported by other studies (Feldman et al., 2010; Schladt et al., 2017). This may be attributable to the technical limitations of ELISA, including issues regarding antibody specificity. The antibody used in the kit may detect not only the free form of the target analyte, but also different forms such as precursors, metabolites, or forms within other protein complexes (Yamamoto and Higashida, 2020). Although these factors may partially explain the differences in the absolute values observed between different ELISA kits and between ELISA and other methods such as HPLC-MS or radioimmunoassay, we were still able to detect physiological changes in oxytocin concentration during pregnancy, labor, and/or lactation using ELISA in blood and saliva samples (Minami et al., 2022). Therefore, although the values may not reflect the absolute physiological values of free oxytocin, the relative change within subjects that we determined is likely to be reliable.

The World Health Organization recently summarized the role of art interventions in improving health and well-being while preventing mental and physical illnesses (Fancourt and Finn, 2019). They emphasized the importance of verifying and evaluating the effectiveness of art activities despite the conceptual challenges in defining “art.” Our study focused on capturing fluctuations in neuroendocrine responses during participatory art workshops. By integrating our physiological measurements with psychological, emotional, or behavioral measurements, we may be able to quantitatively validate the effectiveness of participatory art activities. In future work, we plan to investigate the beneficial effects of these workshops on individuals with ASD and/or other psychiatric disorders. Furthermore, we intend to expand our study to investigate the association between neuroendocrine responses, qualitative measurements, and participant traits to better understand the effectiveness of participatory art activities for different individuals and in various aspects.

## 5 Conclusion

In conclusion, this exploratory study measured the salivary oxytocin and cortisol concentrations in typically developed adults participating in music-making activities using computer software. The neuroendocrinological changes were observed during a music-making workshop. This work highlights the potential value of CSCM activities, although further controlled studies are required to confirm these findings.

## Data Availability

The original contributions presented in the study are included in the article/Supplementary material, further inquiries can be directed to the corresponding author.

## Supplementary material

SUPPLEMENTARY TABLE 1 The number of subjects that participated in either Dyad or Group workshop. The number of participants present at the start time of each workshop is shown. Note 8 participants in the Group workshop starting from 13:10 were pooled from 2 workshops.
