# Supplementary figures and images for: Exploratory study to examine the neuroendocrinological changes in typically developing adults during a music-related participatory practice using computer software
Source: Front Psychol. 2025 Jan 28;15:1431952. doi: 10.3389/fpsyg.2024.1431952 (PMC11810902; doi:10.3389/fpsyg.2024.1431952)

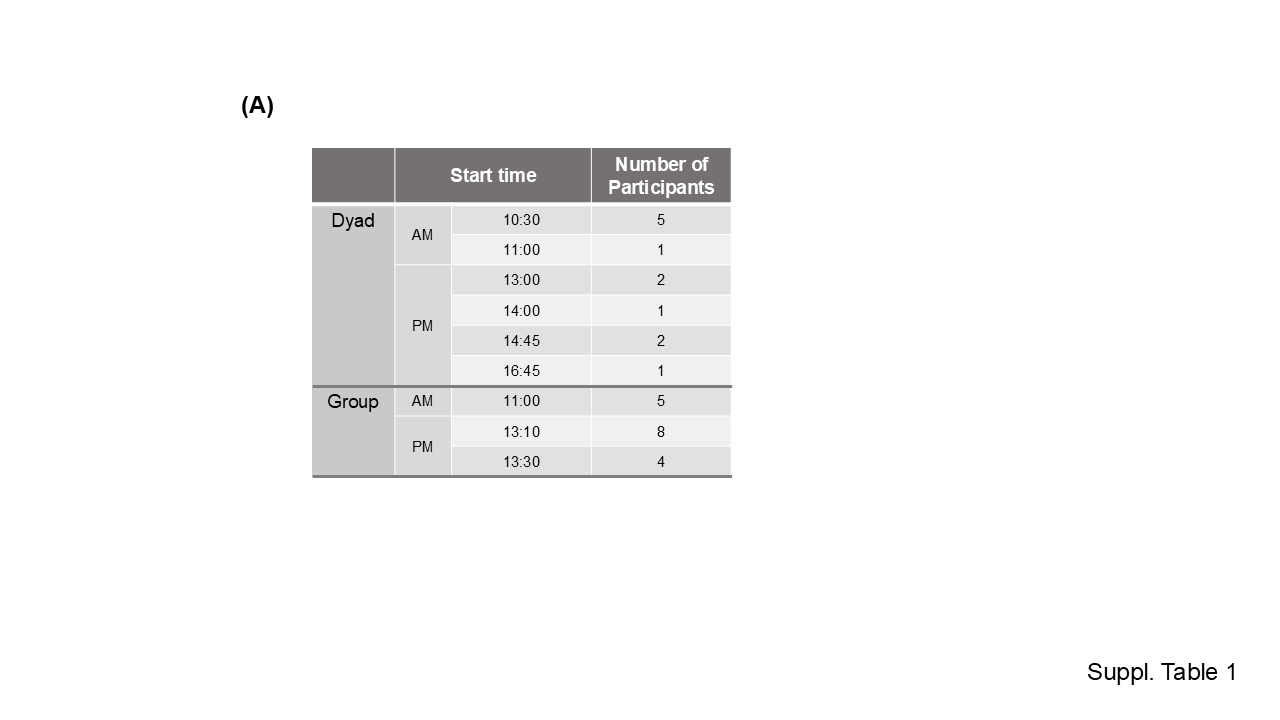

Supplement: SUPPLEMENTARY TABLE 1 — The number of subjects that participated in either Dyad or Group workshop. The number of participants present at the start time of each workshop is shown. Note 8 participants in the Group workshop starting from 13:10 were pooled from 2 workshops. [file Supplementary_file_1.tif]
